# Variable and interactive effects of Sex, APOE ε4 and TREM2 on the deposition of tau in entorhinal and neocortical regions

**DOI:** 10.21203/rs.3.rs-4804430/v1

**Published:** 2024-08-09

**Authors:** Joseph Giorgio, Caroline Jonson, Yilin Wang, Jennifer S. Yokoyama, Jingshen Wang, William Jagust, Alzheimer’s Disease Neuroimaging Initiative, The Health and Aging Brain Study (HABS-HD) Study Team

**Affiliations:** 1 Department of Neuroscience, University of California Berkeley, Berkeley, California, USA, 94720; 2 School of Psychological Sciences, College of Engineering, Science and the Environment, University of Newcastle, Newcastle, New South Wales, Australia, 2308; 3 Center for Alzheimer’s and Related Dementias, National Institutes of Health, Bethesda, MD USA 20892; 4 DataTecnica LLC, Washington, DC, USA, 20037; 5 Pharmaceutical Sciences and Pharmacogenomics Graduate Program, University of California, San Francisco, San Francisco, CA, USA, 94158; 6 Memory and Aging Center, Department of Neurology, Weill Institute for Neurosciences, University of California, San Francisco, San Francisco, CA, USA, 94158; 7 Department of Statistics and Actuarial Science, The University of Iowa, Iowa City, IA, USA.; 8 Department of Radiology and Biomedical Imaging, University of California, San Francisco, CA, USA.; 9 Division of Biostatistics, University of California Berkeley, Berkeley, California, USA, 94720

## Abstract

The canonical AD pathological cascade posits that the accumulation of amyloid beta (Aβ) is the initiating event, accelerating the accumulation of tau in the entorhinal cortex (EC), which subsequently spreads into the neocortex. Here in a sample of over 1300 participants with multimodal imaging and genetic information we queried how genetic variation affects these stages of the AD cascade. We observed that females and APOE-ε4 homozygotes are more susceptible to the effects of Aβ on the primary accumulation of tau, with greater EC tau for a given level of Aβ. Furthermore, we observed for individuals who have rare risk variants in Triggering Receptor Expressed on Myeloid Cells 2 (*TREM2*) and/or APOE-ε4 homozygotes there was a greater spread of primary tau from the EC into the neocortex. These findings offer insights into the function of sex, APOE and microglia in AD progression, and have implications for determining personalised treatment with drugs targeting Aβ and tau.

## Introduction:

For more than a century there were no disease modifying treatments for AD. The recent successful trials of Lecanemab and Donanemab have shown that reducing cortical amyloid-beta plaques (Aβ) led to significant slowing of cognitive decline; however, our grasp on the precise scope and conditions under which anti-amyloid immunotherapy delivers benefits remains unclear ^1,2^. These monoclonal antibodies have been designed based on the amyloid hypothesis, wherein AD follows a canonical cascade, with the accumulation of Aβ being the primary event accelerating tau accumulation and spread from the entorhinal cortex (EC) into the neocortex leading to cognitive decline ^3^. Substantial evidence supports the role of pathological Aβ aggregates as a primary event in both sporadic and genetically dominant forms of AD ^4^. In particular, individuals with dominantly inherited AD mutations show a predictable and sequential evolution of AD pathophysiology with Aβ accumulation leading to tau accumulation and subsequent spread ^5^. However, these genetically dominant forms of AD are rare and the evolution of sporadic late-onset AD is influenced by a multitude of genetic and lifestyle factors, with the relative penetrance of the canonical AD cascade profoundly affected by genetic variation and comorbidities ^6,7^. This is particularly pertinent as both Lecanemeb and Donenamab had varying treatment effects based on the primary outcome in females, and, both Lecanemab and Donanemab showing no significant effects of treatment on the primary outcome in homozygotes for the APOE-ε4 allele ^1 2^. Therefore, it is critical to quantify the impact genetic variation has on the cascading effects of Aβ on entorhinal tau accumulation and subsequent spread into the neocortex to best understand who to treat with these drugs.

The *ε4* allele of *APOE* is the strongest risk factor for clinical sporadic late-onset AD, conferring an increased risk (i.e. odds ratio OR) of AD compared to *APOE-ε3* homozygotes of up to 3.46 OR for *ε4* heterozygotes and 13.04 OR for APOE-ε4 homozygotes ^8^, with APOE-ε4 homozygosity having a near complete penetrance for Aβ positivity ^9^. Interestingly, the APOE-ε4 allele also has a marked increase in the risk of developing AD in Aβ positive individuals, suggesting additional effects beyond Aβ ^10^. Multiple lines of evidence link the APOE4 isoform to reduced homeostatic clearing of Aβ ^6,11–13^. This aberrant function of APOE4 occurs through dysfunction in multiple cell types in the brain that play diverse roles in AD pathogenesis, including neurons, microglia, and astrocytes ^11,14^. Regarding Aβ, APOE4 increases the accumulation of cortical Aβ by reducing the dissolution of soluble Aβ ^15^ and impairs the clearance of Aβ by disrupting the blood brain barrier ^14^. Further, APOE4 has been implicated in increased tau accumulation, with increased levels of APOE4 resulting in increased tau phosphorylation and interneuronal spread of tau ^11,14,16,17^. Therefore, APOE is not only involved in amyloidosis but also in downstream and parallel events such as the development of tau pathology.

Alongside the APOE-ε4 allele, rare genetic polymorphisms in *TREM2* (Triggering Receptor Expressed on Myeloid Cells 2) have also been shown to be significant risk factors for sporadic late-onset AD ^18^. TREM2 is a transmembrane protein expressed in microglia and performs critical functions in the immune response to AD pathology. TREM2 is involved in signalling cascades as well as the transition of microglia to a disease activated state, with a lack of functional TREM2 profoundly impacting microglia function ^19–21^. Multiple *TREM2* polymorphisms have been linked to an increased risk of AD, of which R47H is the most widely studied ^18^. Previous work investigating this variant in animal models shows that rare polymorphisms lead to a hypofunctional form of TREM2 promoting tau seeding and spreading 22. Multiple other genetic variants on the *TREM2* gene have been linked to increased risk of AD and negatively affect the function of TREM2 in-vitro ^18,20,21,23,24^. These variants may have an additive loss of function or alternatively may be in linkage disequilibrium with the same functional variant ^25^. How trait variation in *TREM2* impacts different phases of the AD pathological cascade is yet to be fully elucidated in humans and offers an approach to study how dysfunctional TREM2 impacts microglial functions leading to increased burden of Aβ and tau.

Sex plays an important role in the pathogenesis of AD, with dementia incidence higher in females in late life ^26^. Furthermore, females have consistently been shown to have higher tau tangle load at autopsy then men ^27–29^. This post mortem work is well supported in-vivo with multimodal neuroimaging studies showing females are susceptible to higher levels and faster accumulation rates of tau for a given level of Aβ than their male counterparts ^30–33^. Whether these increases are specific to Aβ influences on primary accumulation of tau in the EC, or tau spreading mechanisms from the EC into the neocortex is not well resolved. Furthermore, it is not clear if this finding in females is due to, or, exacerbated by varied immune responses such as TREM2-related microglial dysfunction.

**Figure 1:**
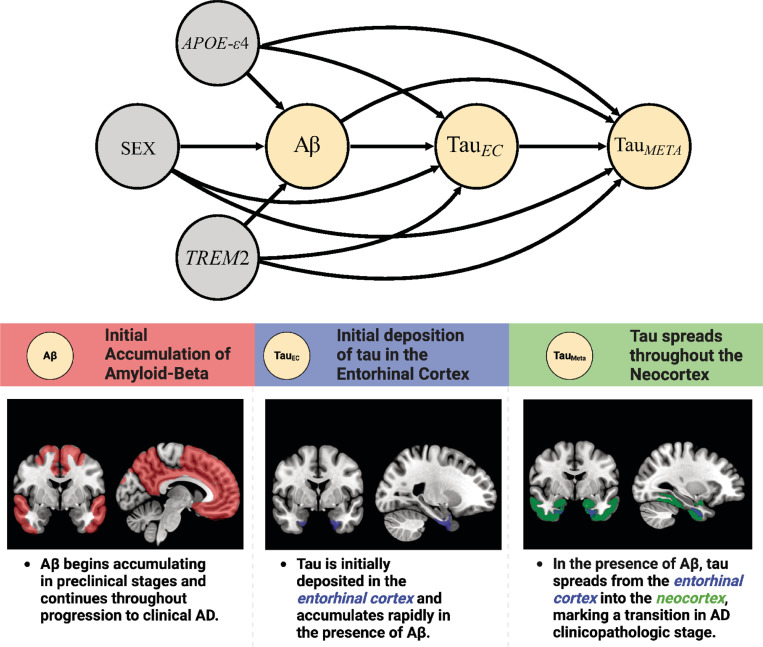
Canonical AD cascade. The top panel shows the directed acyclic graph used to model the potential pathways between genetic variables and pathology. Each arrow represents a potential pathway from a genetic variant (grey node) through an upstream pathology (i.e. Aβ or EC tau) that varies levels of downstream pathology (yellow node). The bottom panel indicates the stages and spatial distribution of AD pathology throughout the cascade. We modelled the initial seeding of tau using the EC region of interest. Early neocortical tau was modelled using the meta temporal region of interest.

Here, we use causal path modelling to assess how genetic variation impacts the AD pathological cascade (Figure 1). Using data from within subject multimodal PET and whole genome sequencing (WGS) in a sample of 1354 individuals we probe different stages of the AD cascade to understand how genetic variation in sex, APOE-ε4 and *TREM2* exacerbate AD pathology. We tested the effect of genetic variation through pathways mediated by Aβ or via non-Aβ pathways, that is, primary tau accumulation in the EC and spread into the neocortex after accounting for Aβ. Our causal path is structured assuming that there are stages in the AD cascade, with the initial event being Aβ deposition, followed by Aβ-related deposition of tau in the EC, followed by tau spreading from the EC into the neocortex (Figure 1).

## Results:

### Participants

We pooled data from the Alzheimer’s Disease Neuroimaging Initiative (ADNI) and Anti-Amyloid Treatment in Asymptomatic Alzheimer’s Disease (A4) study as a discovery sample (n=628, 79% cognitively normal) and drew a racially diverse replication sample (n=726, 76% cognitively normal) from the Health and Aging Brain Study-Health Disparities (HABS-HD) cohort. Participants had varying levels of Aβ, EC tau and neocortical tau as defined using the meta temporal (MetaTemp) ROI ^34^. Furthermore, we extracted genetic information from participants assessing the number of APOE-ε4 alleles in both samples, as well as a binary indicator for *TREM2* risk variant carrier status in the discovery sample. Due to the limited *TREM2* SNPs data in the HABS-HD, we omitted this variable from our replication analysis (**Table 1**).

## Results:

### Causal effects of different genotypes on Aβ.

We first assessed which genetic variants affected levels of Aβ while including age as a confounding variable. Within the discovery sample, we observed significant effects of age (β=0.9650 p<0.0001) and APOE-ε4 (1 allele vs 0:β=27.7510 p<0.0001; 2 allele vs 0:β=41.9344 p<0.0001). We observed no differences in Aβ contrasting sex (Female vs Male: β=−1.2392 p=0.6860) or *TREM2* risk variant carrier status (1 vs 0: β=0.0734 p=0.9905) and a marginally non-significant difference was observed between APOE-ε4 homozygotes and heterozygotes (β=14.183 p=0.08) (**Supplementary Table 1**). Similar results were observed in the HABS-HD sample, with significant effects of age (β=1.16 p<0.0001) and APOE-ε4 (1 allele vs 0:β=11.90 p<0.0001; 2 allele vs 0:β=24.46 p<0.0001). However, we also observed a significant sex effect in this sample (Female vs Male: β=5.58 p=0.004) (**Supplementary Table 2**). We further tested if self reported race confounded these results in the HABS-HD dataset including race as a main effect and interaction term with genetic variables. Both APOE-ε4 and sex remained significant in their effects on Aβ, however their effect sizes differed across different race groups (**Supplementary Table 3**).

### Causal effects of different genotypes on EC-tau.

**Figure 2:**
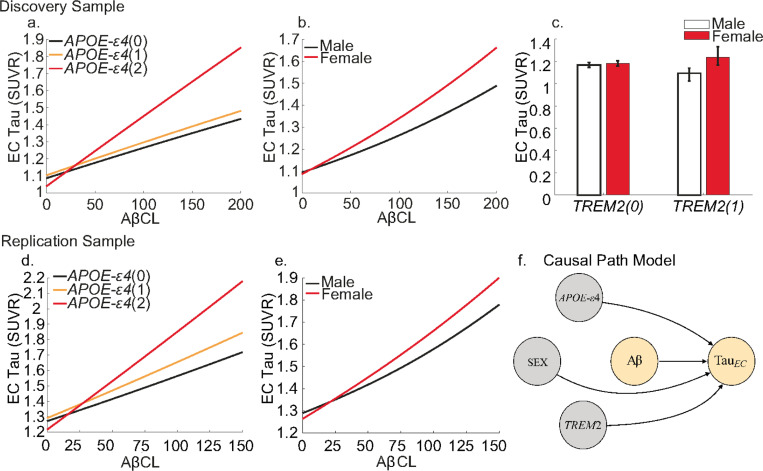
Genetic influences on Entorhinal Cortex (EC) tau. Lines show the estimated marginal levels of tau pathology for individuals with varying genetic profiles at different levels of Aβ. Bar graph shows estimated levels of tau pathology based on sex and *TREM2* risk variant carrier status. Different effects in the discovery sample for **a.** APOE-ε4, **b.** sex, **c.** interaction of sex and *TREM2* risk variant carrier status. Different effects in the replication sample for **d.** APOE-ε4 and **e.** sex. **f.** Causal path model to estimate EC tau. Values in parentheses indicate presence of risk polymorphism or number of risk alleles. The path model shows the causal path modelled whereby levels of EC tau are predicted by genetic variant, Aβ centiloid (CL) and their interactions.

Next, we built a causal path model (Figure 2) to test the main and interactive effects of different genotypes on EC tau (Methods EQ1). Within the discovery sample, we observed significant main effects of Aβ (β=0.0013, P<0.0001) and age (β=0.003, P=0.0072) on EC tau. Furthermore, we observed significant interactions between Aβ and APOE-ε4(2-alleles) (β=0.002, P=0.002), and Aβ and sex(F) (β=0.0008, p=0.032). These interactions indicate that, for a given level of Aβ, APOE-ε4 homozygotes and females had significantly more tau in the EC (Figure 2.). In addition, we observed a significant interaction between *TREM2* and sex, whereby female *TREM2* risk variant carriers had the greatest levels of EC tau (β=0.124 p=0.0407) (**Supplementary Table 4**). Visualising the marginal effects highlights these results showing that APOE-ε4 homozygotes and females have greater EC tau, and these differences increase with Aβ load (Figure 2). We observed highly similar interaction terms in the HABS-HD sample, observing Aβ interacts with APOE-ε4(2-alleles) (β=0.0037, P=0.018) and sex(F) (β=0.0013, p=0.032) (**Supplementary Table 5**). These interactions replicate results showing that for a given level of Aβ, APOE-ε4 homozygotes and females have significantly more tau in the EC (Figure 2.). Our results are likely not confounded by race in the replication sample, as when self-reported race was included as a main effect and interaction term with genetic variables in our model, none of these added variables had a significant influence on EC tau (**Supplementary Table 6**).

### Causal effects of different genotypes on MetaTemp tau.

**Figure 3:**
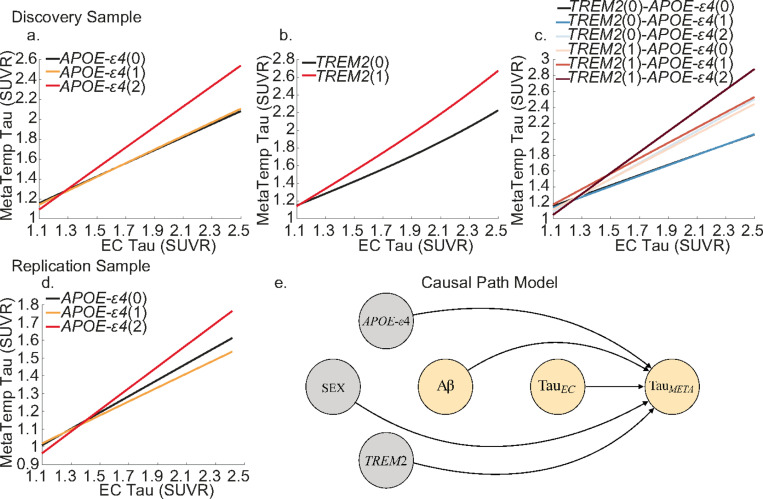
Genetic influences on MetaTemp tau. Lines show the estimated marginal levels of MetaTemp tau pathology for individuals with varying genetic profiles at different levels of EC tau. Different effects in the discovery sample for **a.** APOE-ε4, **b.** *TREM2*, and **c.** their interaction. Different effects in the replication sample for **d.** APOE-ε4. **e.** causal path modelled whereby levels of MetaTemp tau are predicted by genetic variant, Aβ centiloid, EC tau and their interactions.

We then built a causal path model (Figure 3) to test the main and interactive effects of different genotypes on MetaTemp tau (Methods EQ2). To allow for an accurate estimation of the causal effects when two continuous variables interact, we segmented Aβ into four bins (<10CL, 10–40CL, 40–60CL and >60CL) when including it as an interactive term with EC tau. This Aβ variable is then used as a candidate variable when selecting interaction terms for the causal path model for MetaTemp tau. Continuous Aβ is still included as a main effect variable in model selection. Within the discovery sample, we observed significant main effects of EC tau (β=0.364, P<0.001), *TREM2* risk variant carrier status (β=−0.3580 p=0.0001) and APOE-ε4 homozygosity (β=−0.3580 p=0.0001) on MetaTemp tau. In addition, we observed significant interactions between EC tau and high Aβ (>60CL) (β=0.019 p=0.049), EC tau and APOE-ε4(2-alleles) (β=0.364, P<0.001), and EC tau and *TREM2*(1) (β=0.309, P<0.001). Individuals with high levels of Aβ, APOE-ε4 homozygotes and individuals with a *TREM2* risk variant carrier status having greater downstream MetaTemp tau burden for a given level of EC tau (Figure 3). A significant interaction between APOE-ε4 and *TREM2* (β=0.0186, p=0.0168) indicated that those with a *TREM2* risk variant and 1 or 2 APOE-ε4 alleles had significantly more MetaTemp tau. Visualising the marginal effects highlights these results showing that APOE-ε4 homozygotes and *TREM2* risk variant carriers have greater MetaTemp tau, and these differences increase with EC tau burden, independent of Aβ main and interactive effects with EC tau (Figure 3). We did not observe any significant effects of sex on MetaTemp tau (sex(F) (β=0.0613, p= 0.1407); sex(F)*EC tau (β=−0.0504, p= 0.1496)) (**Supplementary Table 7**). Where possible due to available data, we observed highly similar results in the HABS-HD sample. We observed significant effects of APOE-ε4 homozygosity on MetaTemp tau both as a main effect (β=−0.216 p=0.0069) and interacting with EC tau burden (β=0.156, P=0.0047) (Figure 3). This replicates the association we observed showing that APOE-ε4 homozygotes have greater MetaTemp tau, and these differences increase with EC tau burden independent of Aβ effects. Similar to the discovery sample, we did not observe any significant effects of sex on MetaTemp tau (sex(F) (β=−0.013, p= 0.74); sex(F)*EC-tau (β=0.0305, p= 0.31)) (**Supplementary Table 8**). Like the previous analysis for EC tau, our results are likely not confounded by race in the replication sample. When self-reported race was included as a main effect and interaction term with genetic variables in our model, these added variables did not have significant influences on MetaTemp tau (**Supplementary Table 9**).

### Variable genetic effects at different stages in the canonical amyloid cascade pathway.

Finally, we investigated the variability in the direct, mediation, and total effects of upstream pathology on downstream tau pathology for different genetic profiles. This allows us to probe each aspect of the AD cascade and estimate which groups are likely to have higher downstream pathology for a given level of upstream pathology (i.e. if a population has greater EC tau for a given level of Aβ and how this may result in higher levels of MetaTemp tau). To do this, in each group we calculate the direct effect of Aβ on EC tau (${\text{DE}}_{\text{Aβ ->EC tau}}$); the direct effect of EC tau on MetaTemp tau (DE_EC tau ->MetaTemp tau_); the direct effect of Aβ on MetaTemp tau (${\text{DE}}_{\text{Aβ ->MetaTemp tau}}$); the mediation effect of Aβ through EC tau on MetaTemp tau (${\text{ME}}_{\text{Aβ ->EC-tau -> MetaTemp tau}}$); and finally the total effect of Aβ on MetaTemp tau (${\text{TE}}_{\text{Aβ -> MetaTemp tau}}$). We observed significant effects in all AD pathways except the ${\text{DE}}_{\text{Aβ ->MetaTemp tau}}$ regardless of genetic profile due to ${\text{ME}}_{\text{Aβ ->EC-tau -> MetaTemp tau}}$ being consistently significant. That is, in these data the role of Aβ on MetaTemp tau is via the canonical pathway acting through increases in EC-tau. When we contrast these effects amongst the different genetic groups, we observe differences in the total effects of Aβ on MetaTemp between APOE-ε4 homozygotes, where this population has significantly greater levels of MetaTemp tau for a given level of Aβ ${\text{TE}}_{\text{Aβ -> MetaTemp tau}}$: APOE-ε4 (2-alleles) vs (0-alleles)=0.003,p=0.022; (2-alleles) vs (1-allele)=0.0029,p=0.0335)). This is due to differences in both ${\text{DE}}_{\text{Aβ ->EC tau}}$ and DE_EC tau ->MetaTemp tau_ for APOE-ε4 homozygotes (**Supplementary Table 10**). This implies that for APOE-ε4 homozygotes, a given level of Aβ leads to higher EC-tau, and a given level of EC-tau leads to higher MetaTemp tau. As such, this population sees greater levels of MetaTemp tau for a given level of Aβ due to increased effects of Aβ on EC tau aggregation and EC tau spread into the neocortex. There were no significant differences in the ${\text{TE}}_{\text{Aβ -> MetaTemp tau}}$ across other genetic groups, although of note was a non-significant numerical difference in the ${\text{TE}}_{\text{Aβ -> MetaTemp tau}}$ for females vs males (females vs males=0.0005, p=0.1), which was driven by significant differences in the ${\text{DE}}_{\text{Aβ ->MetaTemp tau}}$ (females vs males=0.0008, p=0.036). This implies that for a given level of Aβ women have greater levels of EC tau, although, this doesn’t require that females have reliably greater MetaTemp tau for the same level of Aβ. Relative effects and interpretations were similar in the replication sample (**Supplementary Table 11**).

## Discussion:

Here we used causal path analyses structured on the canonical AD pathological cascade where Aβ is the initiating event, followed by increased tau burden in the EC, followed by tau involvement of neocortex. Using this path framework, we examined how genetic variation relates to the burden of tau pathology in the medial temporal and neocortex. We provide compelling evidence for heterogeneity in how regionally specific tau pathology is distributed based on different genetic traits, thus providing insight into the biological mechanisms that may govern increased tau pathology. Furthermore, we provide empirical evidence that may explain variable gene and sex related treatment effects of recent anti-amyloid immunotherapy trials.

Using rare polymorphisms on the coding region of the *TREM2* gene, we find that trait differences in the function of TREM2 plays a role in the spread of tau from the EC into the neocortex. We observed a strong effect showing that for a given level of tau in the EC, individuals with a gene burden of *TREM2* have greater levels of neocortical tau after accounting for other upstream and confounding variables (i.e. sex, APOE-ε4, age, Aβ). The function of TREM2 has been linked to tau spread through several putative mechanisms ^18,19^. In particular, animal models have highlighted that *TREM2* risk variants result in hypoactive TREM2 which may disrupt microglial inflammatory signalling to tau and may promote tau transmission through aberrant microglial activity ^23,35–38^. Prior neuroimaging studies in humans has similarly implicated microglial activity in tau deposition in the neocortex ^39^. Furthermore, CSF soluble TREM2 (sTREM2) levels are predictive of the transition from pre-clinical to clinical AD and are associated with CSF tau levels ^18^. Alternate accounts have also shown that increased CSF levels of sTREM2 may be protective against tau and Aβ accumulation ^40^. Our work adds to this body of literature by interrogating the function of TREM2 as a parent trait variable rather than an indicator which varies as a function of pathological state, providing in-vivo evidence into how dysfunction in TREM2 enters the AD cascade, working to exacerbate tau spread from the EC into the neocortex likely through aberrant microglial function.

We observe strong effects of APOE-ε4 homozygosity on both tau in EC and neocortex. Specifically, across levels of Aβ APOE-ε4 homozygotes had substantially more EC-tau. Further, for a given level of EC-tau APOE-ε4 homozygotes had greater levels of neocortical tau. Critically, this relationship between APOE-ε4 homozygosity and variable neocortical tau burden was observed after accounting for the levels of upstream Aβ, and the interaction between high Aβ and EC tau. The net result of these effects means that for a given level of Aβ, APOE-ε4 homozygotes have a greater level of neocortical tau than non-carriers and heterozygotes. Multiple lines of evidence have shown that overexpression of APOE4 increases tau phosphorylation ^16,41^ and spread of tau ^11,14^. Prior evidence from APOE-ε4 models shows that the activity of neuronal ^42,43^, astrocytic ^44^ and microglial ^45,46^ cells increases tau pathology. Furthermore, APOE-ε4 knock in mice have displayed hyperexcitability in medial temporal regions ^43,47^, a potential driver of tau accumulation ^48^. Converging human neuroimaging studies have implicated the APOE-ε4 allele with increased levels of medial temporal tau independent of Aβ ^49–52^. Our work builds on these previous accounts showing that APOE4 also plays a role in tau deposition outside of the EC and through tau mediated routes. Of note, these previous studies generally grouped APOE-ε4 carriership as a binary variable and did not contrast homozygotes with heterozygotes. We observe that it is homozygotes that are more susceptible to increased levels of tau after accounting for Aβ. Given the substantially greater risk of having clinical AD ^8^ and Aβ positivity ^9^ in homozygotes compared to heterozygotes, this supports recent work showing that statistical aggregation of APOE-ε4 homozygotes and heterozygotes in one group may not be appropriate ^9^. Although we were well powered to make comparisons between APOE-ε4 homozygotes and heterozygotes, further work in larger samples will be required to fully understand the differences in tau accumulation between these groups. Recent imaging studies have suggested that APOE-ε4 potentiates the relationship between Aβ and tau pathologies ^53,54^; our results provide some support of this model, highlighting that Aβ and APOE-ε4 interact to increase EC tau pathology. However, we suggest a refinement to this model whereby APOE-ε4 also potentiates the relationship between tau burden in the EC and spread into the neocortex independent of Aβ.

We have shown that APOE-ε4 homozygotes have increased primary tau pathology in the EC at lower levels of Aβ, and increased neocortical tau pathology at lower levels of EC tau. This strongly suggests that homozygotes should be treated earlier with anti-amyloid treatment (i.e. at lower levels of Aβ) to reduce tau, not simply because they begin depositing Aβ earlier than other genotypes ^55^ but because this Aβ drives higher tau. In addition, the effects of homozygosity on increased neocortical tau relative to EC tau raise questions about the need for anti-tau therapy in this group. This empirical evidence may provide some of the biological underpinnings explaining lack of treatment effect in APOE-ε4 homozygotes in both the Lecanemab and Donenamab trials ^1 2^.

In addition to the independent effects of *TREM2* and APOE-ε4, we observed a significant interaction between these two genetic risk traits. Individuals with a *TREM2* risk variant and an APOE-ε4 allele had higher levels of neocortical tau for a given level of EC tau, implicating interactive factors between APOE4 and TREM2 on tau aggregation. APOE is a ligand of TREM2, with models of *TREM2* variants (*R47H, R62H, D87N*) showing a decreased binding affinity between TREM2 and APOE in vitro ^19,56–58^. Furthermore, prior work shows that APOE4 isoforms are recognised and engulfed by TREM2 at different rates than other APOE isoforms ^59^; this differential binding of TREM2 to APOE4 may result in an impaired switch of homeostatic microglia to disease associated microglia in AD ^18,20,21^. Previous neuroimaging studies have also implicated the APOE-ε4 allele as a modulating factor between microglial activity and tau spread independent of Aβ ^49^. Our results support this previous human work highlighting that genetic traits that may manifest in aberrant interactions between TREM2 and APOE4 likely have a profound effect on the spreading of tau from the EC into the neocortex.

After accounting for upstream pathologies, we observed that females who harbor a risk variant in *TREM2* have higher levels of EC tau after accounting for all other upstream or confounding variables (i.e. Aβ, APOE-ε4 and age). Previous work has pointed to interactive factors of microglial activity and sex, implicating a greater role of microglia in tauopathy for females ^60^. Here we show similar trait interactions between the risk gene burden of *TREM2* and female sex in EC tau after accounting for upstream variables. In addition, when investigating how sex interacts with Aβ we observe that females have higher levels of EC tau for a given level of Aβ than their male counterparts. This result fits well with previous accounts showing females have higher levels of medial temporal lobe tau after controlling for Aβ ^31–33^. We did not observe significant effects of sex on tau in neocortical regions suggesting that females are more susceptible to early tau deposition in the EC but may not show differences in tau spreading into the neocortex. This is further reflected in the small differences between females and males in the total effect of Aβ on MetaTemp tau, which is predominantly driven by a larger direct effect of Aβ on EC-tau in females. This suggests that females should see similar benefits in anti-amyloid treatment as males but may require treatment at lower levels of Aβ --so as to be at similar levels of primary tau-- or to be screened for both Aβ and tau, ensuring that females do not have more advanced tau pathology than their male counterparts. These findings may also provide some insights into the discrepancies between the Lecanemab and Donenanab trials, whereby females did not see the same benefit as males in Lecanemab but did in Donenanab ^1 2^. It is feasible that the multimodal screening for intermediate tau and Aβ positivity in Donenanab vs. unimodal screening of Aβ positivity in Lecanemab ensured that the sex related differences in early tau burden were ameliorated. Confidence in such an interpretation will require further exploration however.

Our interpretation and approach have several limitations. First, we note that our interpretation of our results in light of the recent anti-amyloid trials is highly susceptible to confirmation bias. Although we initiated this work prior to the release of the results of these trials, we did undertake much of the preparation of this manuscript knowing that APOE-ε4 homozygotes and females may have variable treatment outcomes. Although our results fit well in existing literature, further work is required to understand the reasons that these populations saw attenuated or no benefit in the respective trials. Second, although our model is grounded in generally well accepted neuropathological staging of AD ^61,62^, it is cross sectional so further modelling work on longitudinal multimodal imaging datasets is required to fully elucidate the dynamics of AD pathophysiological changes. Third, our analytical decision to consolidate multiple *TREM2* risk variants into a risk variant carrier status assumes a similar biological role of each risk variant and does not apply a weighting to individual SNPs ^63^. Furthermore, the 6 SNPs were not fully sampled in each cohort with 4 of 6 sampled in ADNI and some variants were imputed from the WGS in A4. Therefore, it is possible that some individuals who have a *TREM2* risk variant were assigned to the non-carrier group due to lack of available data. However, both of these analytical decisions would work to push our results closer to the null (i.e. more prone to type II error) and thus do not negate the findings presented here. Future work on larger and more complete datasets will afford a more granular appraisal of the biological role of each risk SNP on the AD cascade. Similarly, although we validated our findings related to sex and APOE-ε4 in an ancestrally diverse sample, we were unable to undertake robust ancestral stratification to fully understand potential ancestral differences that are observed in AD genetic risk ^8^. However, we do note that there was a reasonable representation of each ethnicity in our validation sample of APOE-ε4 homozygotes. Further, although our sample is large, the number of APOE-ε4 homozygotes is still small. However, we did replicate our findings in two independent and relatively heterogeneous samples, giving further confidence to our findings regarding APOE-ε4. Finally, due to the incomplete sampling of *TREM2* SNPs in HABS-HD, we were unable to run a replication analysis and as such further work will be required to validate our *TREM2* findings.

## Conclusion:

Our genetic architecture at birth governs the function of our biological systems throughout development, ageing, and neurodegeneration. Here we have used genetic variation to provide a deeper understanding of the biological mechanisms that drive AD pathophysiology. We show in a diverse sample of over 1300 participants that females and APOE-ε4 homozygotes are more susceptible to the primary accumulation of tau, with greater EC tau for a given level of Aβ. Furthermore, we observed for individuals with risk variants in *TREM2* and APOE-ε4 homozygotes there was a greater spread of primary tau from the EC into the neocortex. These findings offer insights into the function of sex, APOE and microglia (vis a vis TREM2) in AD progression and have implications for determining personalised treatment with drugs targeting Aβ and tau.

## Methods:

### Participants

We pooled two well‐characterised ageing and AD cohorts as a discovery sample (n=628, 79% cognitively normal). A sample of clinically impaired and cognitively normal (n=297, 55% cognitively normal) participants were drawn from the Alzheimer’s Disease Neuroimaging Initiative (ADNI) and were combined with a sample of cognitively normal (n=331) participants drawn from the screening visit of the Anti-Amyloid Treatment in Asymptomatic Alzheimer’s Disease (A4) study. We selected participants from their respective studies who had whole genome sequencing (WGS), tau and Aβ PET imaging. This restricted the A4 sample to predominantly represent the elevated amyloid group with a small subset from the Aβ not elevated LEARN observational study. The majority of participants within this discovery sample were self-described as White (93%). In addition, we drew a racially diverse replication sample (n=726) from the Health and Aging Brain Study-Health Disparities (HABS-HD) cohort, selecting participants who had tau, Aβ PET imaging, and APOE-ε4 genotyping. This sample was predominantly cognitively normal (76%) and well balanced for racial diversity amongst White (38%), Hispanic (30%) and Black (32%) populations (**Table 1**).

### Neuroimaging

#### FTP (Flortaucipir PET) Tau

The ADNI [^18^F] Flortaucipir (FTP) FTP-PET protocol entailed the injection of 10 mCi of FTP followed by acquisition of 30 min of emission data from 75–105 min post injection. The A4 FTP-PET protocol acquired 30 min of emission data from 80–110 min post injection. The HABS-HD [^18^F] PI-2620 tau PET protocol entailed the injection of 10 mCi of PI-2620 followed by the acquisition of 30 min of emission data from 45–75 minutes.

Tau PET data were realigned, and the mean of all frames was used to coregister tau PET to each participant’s MRI acquired closest to the time of the tau PET. Tau PET standardised uptake value ratio (SUVR) images were normalised to inferior cerebellar grey matter. MR images were segmented and parcellated into the Desikan-Killiany atlas using Freesurfer (V5.3) and regions of interest were used to extract cerebellar-normalised regional SUVR data. SUVR data was summarised for two regions of interest (ROIs) in the entorhinal cortex and the tau meta temporal (MetaTemp) ROI, comprised of the volume weighted average of the entorhinal, amygdala, parahippocampal, fusiform, inferior temporal, and middle temporal ROIs. Due to variable scanner resolution in the A4 FTP dataset, the smoothness of data for each scan site could not be reliably estimated for all subjects, therefore we did not partial volume correct any dataset. All tau PET data were analysed using the same in-house pipeline at UC Berkeley and SUVR values are used throughout as a tracer specific harmonised scale for EC tau and MetaTemp tau.

#### Aβ PET imaging

ADNI Aβ imaging was performed at each ADNI site using either [^18^F] Florbetapir (FBP) or [^18^F] Florbetaben (FBB). A4 Aβ imaging was performed at each site using FBP. HABS-HD Aβ imaging was performed using FBB. The FBP Aβ-PET protocol involved injection of 10 mCi of FBP followed by the acquisition of 20 min of emission data at 50–70 min post injection. The FBB protocol involved injection of 8.1 mCi of FBB followed by 20min of emission data at 90–110min post injection. Aβ-PET images were then processed to derive a summary of global Aβ burden in Centiloids (CL) that are used throughout as a harmonised scale for Aβ-PET burden ^64^. The CL conversion for the ADNI and HABS-HD samples was performed using in-house processing pipelines at UC Berkeley, A4 CL values were downloaded from LONI.

### Genetics:

#### TREM2.

We selected 6 rare variant single nucleotide polymorphisms (SNPs) on the coding region of the *TREM2* gene that have previously been associated with increased AD risk *rs2234256, rs2234255, rs142232675, rs143332484, rs75932628, rs2234253* ^23,65–69^.

Genetic data for ADNI and A4 were downloaded from The National Institute on Aging Genetics of Alzheimer’s Disease Data Storage Site (NIAGADS DSS) and the Laboratory of Neuro Imaging Image and Data Archive (LONI IDA), respectively. ADNI genetic data was whole genome sequencing data from ADSP’s August 15, 2022 release in the form of a VCF file. A4 data were Plink files, filtered for non-Hispanic white (NHW) individuals imputed on the TOPMed imputation server. BCFTOOLs was used to extract our 6 variants of interest. We interrogated the SNP data for the HABS-HD dataset and found only 2 of the 6 *TREM2* variants were sampled.

We collapsed across the 6 SNPs to form a binary *TREM2* risk variant carrier status ^63^, where the presence of any of the a-priori SNPs was labelled a 1 and the absence was labelled a 0. There were no weightings applied to the SNPs. Due to the limited *TREM2* SNPs data in the HABS-HD, we omitted this variable from our replication analysis. The number of APOE-ε4 alleles for each participant was downloaded from LONI for the A4 and ADNI samples. For the HABS-HD data APOE-ε4 data was made available through the University of North Texas Institute for translational research. For all studies, individuals were classified as APOE-ε4 non-carriers, heterozygotes, and homozygotes (**Table 1**).

### Statistical analysis:

#### Path analysis under the amyloid cascade hypothesis.

Using Structural equation models (SEM) with interacting terms we built the possible impacts that genetic parent variables (sex, APOE-ε4, *TREM2*) have on different aspects of the AD pathological cascade, including age as a predictor to account for its potential confounding effect. We built the path following the canonical amyloid cascade hypothesis whereby Aβ is the initiating event, followed by increased levels of tau in the EC, then increased levels of tau in the neocortex (i.e. MetaTemp ROI) (Figure 1). We assessed each stage of the AD cascade by building a series of SEMs modelling all main and 2-way interactions between genetic parent variables and upstream pathologies. To ensure a parsimonious description of the data we employed forward variable selection in each SEM based on Akaike Information Criterion (AIC). To allow for an accurate estimation of the causal effects when two continuous variables interact (i.e. Aβ and EC tau), we discretise Aβ into four bins (< 10, 10–40, 40–60, > 60) when including it as an interactive term with EC tau. Continuous Aβ is still included as a main effect variable when interactions are retained following model selection. Following variable selection the following reduced models were investigated.


EQ0 $$A\beta =\mu_{\text{0}}+\alpha_{\text{0}}\text{Age}+\beta_{\text{0}}\text{Sex}+\gamma_{\text{0}}\;\text{TREM2}+\delta_{01}{\text{APOE4}}_{1}+\delta_{02}{\text{APOE4}}_{2}+\epsilon_{0}$$

EQ1 $$\tau_{\text{EC}}=\mu_{1}+\theta_{\text{1}}A\beta +\alpha_{\text{t}}\text{Age}+\beta_{1}\text{Sex}+\gamma_{\text{1}}\text{TREM}2+\delta_{\text{11}}{\text{APOE4}}_{1}+\delta_{\text{12}}{\text{APOE4}}_{2}+\lambda_{11}A\beta \times {\text{APOE4}}_{1}+\lambda_{12}A\beta \times {\text{APOE4}}_{2}+\;\;\zeta_{1}A\beta \times \text{Sex}+\phi_{1}\text{Sex}\times \text{TREM2}+\epsilon_{1}$$

EQ2 $$\tau_{\text{Meta}}=\mu_{2}+\theta_{2}A\beta +\kappa_{2}\tau_{\text{EC}}+\alpha_{2}\text{Age}+\beta_{2}\text{Sex}+\gamma_{2}\text{TREM2}+\delta_{21}{\text{APOE4}}_{1}+\delta_{22}{\text{APOE4}}_{2}+v_{2}1_{\left\{A\beta \geq 60\right\}}\times \tau_{\text{EC}}+\tau_{2}\tau_{\text{EC}}\times \text{TREM}2+\pi_{21}\tau_{\text{EC}}\times \;\;{\text{APOE4}}_{1}+\pi_{22}\tau_{\text{EC}}\times {\text{APOE4}}_{2}+\omega_{21}\text{TREM2}\times {\text{APOE4}}_{1}+\omega_{22}\text{TREM2}\times {\text{APOE4}}_{2}+\;\;\psi_{2}A\beta \times \text{TREM2}+\eta_{2}\tau_{\text{EC}}\times \text{Sex}+\epsilon_{2}.$$

To assess the statistical differences between estimated effects for different levels of genetic variables we ran models on 1000 bootstrapped samples. When genetic factors interact with upstream pathologies, we visualise these results by showing the marginal effects across different levels of upstream pathologies. In the R version 4.1.1 environment, we applied the SEMs to the combined dataset, excluding two outliers (RIDs 4414 and 4715 from the ADNI dataset) identified through diagnostic checks. Using the parameter estimates obtained from the SEMs, we calculated the marginal effects by subsequently varying the levels of genetic factors, Aβ (in EQ1), and EC tau (in EQ2). We then fit the HABS-HD data to the reduced models to assess if the estimates observed in the discovery data replicate to a new sample. Due to incomplete *TREM2* information in the HABS-HD we omitted this variable from replication models.

Finally, we calculated the direct, mediation and total effects along the pathway from Aβ to EC tau to MetaTemp tau for different levels of genetic variables, contrasting levels of downstream pathology (i.e. MetaTemp tau) for a given level of upstream pathology (i.e. Aβ) under varying genetic profiles. The mediation effect was quantified by incrementing Aβ by one unit in the EQ1, observing the resultant change in EC tau, and then applying this change to the EQ2 to measure the consequent variation on MetaTemp tau. The detailed derivations of the mediation effect are shown in **Supplementary Methods - Mediation Effect Analysis**. We also calculated the sample size weighted mean of the mediation effect for varying levels of *APOE4*, *TREM2*, and sex to elucidate the mediation effect with respect to individual factors.
